# Identification and Antioxidant Capacity of Free and Bound Phenolics in Six Varieties of Mulberry Seeds Using UPLC-ESI-QTOF-MS/MS

**DOI:** 10.3390/antiox11091764

**Published:** 2022-09-07

**Authors:** Huaqi Gao, Meimei Guo, Liqin Wang, Cui Sun, Lingxia Huang

**Affiliations:** 1 College of Animal Sciences, Zhejiang University, Hangzhou 310058, China; 2 Key Laboratory of Silkworm and Bee Resource Utilization and Innovation of Zhejiang Province, Hangzhou 310058, China; 3 Linyi Forestry Bureau of Shandong Province, Linyi 276003, China; 4 Institute of Fruit Science, College of Agriculture and Biotechnology, Zhejiang University, Hangzhou 310058, China

**Keywords:** mulberry seeds, free phenolics, bound phenolics, UPLC-ESI-QTOF-MS/MS, antioxidant capacity

## Abstract

Mulberry seeds are a byproduct of juice processing and may be an important resource for its abundant compounds. In this study, we analyzed the qualitative composition of free and bound phenolics from six varieties of mulberry seeds using UPLC-ESI-QTOF-MS/MS. Free phenolics (FPs) and bound phenolics (BPs) were measured using the Folin–Ciocalteu method; antioxidant capacity was determined by measuring 2,2-diphenyl-1-picrylhydrazyl radical-scavenging activity, using the ferric reducing antioxidant power assay. A total of 28 free and 11 bound phenolics were extracted and identified, wherein five free phenolics were found in mulberry matrices for the first time. The six varieties of mulberry seeds exhibited higher content of FPs than BPs, and there was a correlation between the phenolic content and antioxidant capacity. Consequently, three varieties were selected for their high phenolic content and antioxidant capacity. This study might offer a theoretical basis for the utilization of mulberry seed.

## 1. Introduction

Mulberry (*Morus alba* L.) is an important plant from the Moraceae family, widely cultivated under different climatic conditions around the world, including China and India [1]. Studies on the various types and parts of the mulberry plant, including its fruits, leaves, branches, and Mori Cortex, have increased. Previous studies have shown that mulberry is abundant in bioactive compounds including flavonoids, carotenoids, anthocyanins, polysaccharides, alkaloids, stilbenes, and diels-alder type adducts [2,3,4], which provide the mulberry plant with a variety of biological properties including antioxidant, antibacterial, anti-inflammatory, hepatoprotective, antidiabetic, and anti-tumor activities [5,6].

The fruit of the mulberry contains a seed in every ovary [7]. Mulberry seeds may be obtained from ripe fruits and are a byproduct of juice processing. Each year, total mulberry production exceeds 6.5 million tons in China; therefore, mulberry seeds are available in tremendous quantities in the food industry [8]. However, compared with other mulberry matrices, mulberry seeds have attracted less attention. Gecgel et al. [9] found that 100 g of mulberry seeds were comprised of 27.5–33% crude oil, 20.2–22.5% crude protein, 3.5–6% ash, 42.4–46.6% carbohydrates, and 112.2–152.0 mg total phenolics, indicating that it is a rich source of bioactive substances. Given the high oil content (around 30–40%), there have been some studies on the composition [10] and antioxidant activity [11] of mulberry seed oil, in addition to the novel lipids it contains [8].

Phenolics in the leaves of mulberry fruits have been reported in several studies. Moreover, they have proven to be a vital component of mulberry seeds [9] and are usually free or bound to the cell wall of the seeds. Free phenolics (FPs) can be extracted using various solutions, such as water, methanol, and ethanol, whereas bound phenolics (BPs) are insoluble and closely associated with the structural components of the cell wall [12]. Several studies have reported that mulberry seed extract is rich in phenolics, such as γ-tocopherol and ellagic acid derivatives [11,13], with caffeic acid, 3,4-dihydroxybenzoic acid, rutin, and cyanidin-3-rutinoside as the major phenolics [14]. However, in comparison with other mulberry matrices, such as leaves and fruits, fewer researches have been conducted on phenolics in mulberry seed and studies on comprehensively identifying free and bound phenolics in mulberry seeds have not been reported.

In the present study, six varieties of mulberry seed in China were selected including Shisheng, Yu 711, Guiyou 12, Guiyou 62, Teyou 2, and Yue 69851. Free and bound phenolics were extracted by methanol and ethyl acetate, respectively, and analyzed by ultra performance liquid chromatography coupled with electrospray quadrupole time-of-flight mass spectrometry (UPLC-ESI-QTOF-MS/MS). The free and bound phenolic content and antioxidant activity were measured using 2,2-diphenyl-1-picrylhydrazyl (DPPH) and the ferric reducing antioxidant power (FRAP) assay. The results of this study provide the first comprehensive analysis of FPs and BPs in mulberry seeds and a basis for the development and utilization of bioactive substances from mulberry seeds.

## 2. Materials and Methods

### 2.1. Chemicals and Reagents 

All chemicals and reagents used were of analytical grade. The water was double distilled (ddH_2_O). Methanol and acetonitrile of chromatographic purity were purchased from Sigma-Aldrich (St. Louis, MO, USA). N-hexane, ethyl acetate, gallic acid, NaOH, Folin–Ciocalteu, Na_2_CO_3_, Trolox, 2,2-diphenyl-1-picrylhydrazyl (DPPH), FeCl_3_, HCl, NaAc, and 2,4,6-tris (2-pyridyl)-s-triazine (TPTZ) were purchased from Sinopharm Chemical Reagent Co., Ltd. (Shanghai, China).

### 2.2. Standards and Standard Solutions

The standards of the chemicals used were of chromatographic grade, (≥98%). For identification purposes, tryptophan, rutin, isofraxidin, kaempferol-3-o-rutinoside, coniferaldehyde, isochlorogenic acid, hesperidin, 3,4-dihydroxybenzoic acid, *P*-hydroxybenzoic acid, vanillic acid, caffeic acid, vanillin, *P*-coumaric acid, and ferulic acid were purchased from Shanghai Yuanye Bio-Technology Co., Ltd. (Shanghai, China).

### 2.3. Samples

Six varieties of mulberry seeds were used for this study. Guiyou 12, Guiyou 62, and Teyou 2 were purchased from the Guangxi Nanning Tianlong Biological Technology Co., Ltd. (Guangxi, China). Yue 69851 and Yu 711 were purchased from Guangdong Siji Mulberry Garden Sericulture Technology Co., Ltd. (Guangdong, China). Shisheng was obtained from Zhejiang Haining Sericulture Technology Research Institute (Zhejiang, China). The mulberry seeds were ground to pass through a 60-mesh sieve and stored at −20 °C until further analysis.

### 2.4. Extraction of Free Phenolics (FPs) 

Mulberry seed powder was defatted using *n*-hexane. The extraction process for FPs was optimized based on a previous procedure [15,16]. One gram of defatted mulberry seed powder was mixed at a ratio of 1:5 (*m*/*v*) with 80% methanol and extracted in an ultrasonic bath for 40 min. The supernatants were centrifuged at 3000 rpm for 7 min, collected, then 4 mL of 80% methanol was added, and the extraction procedure was repeated two times. The supernatants were collected and filtered through a 0.45 μm organic filter membrane. The methanol extracts were dissolved in water to obtain the sample solution after removing the organic solvent by vacuum rotary evaporation.

Solid phase extraction was used to separate the phenolics from the methanol extracts. C18 Sep-Pak cartridges (Agilent, Santa Clara, CA, USA) were preconditioned with 12 mL of methanol and 24 mL of ddH_2_O. The sample solution was passed through the cartridge, which was washed with 60 mL of ddH_2_O to remove impurities, such as sugar and acid. The absorbed phenolics were eluted with 24 mL of methanol. The methanol in the eluent was removed by vacuum rotary evaporation at 37 °C, and the residue was dissolved with a small amount of ddH_2_O. The methanol extract powder was obtained by vacuum freeze drying of the aqueous solution. The power was dissolved in methanol and the solution was filtered through a 0.45 μm organic filter membrane for UPLC-ESI-QTOF-MS/MS.

### 2.5. Extraction of Bound Phenolics (BPs)

The extraction process was based on the procedure by Singh et al. [17], with a few modifications. The residue after methanol extraction was hydrolyzed with 15 mL of 2 mol/L NaOH for 4 h in the dark. Then, the mixture was acidified to pH = 2.0 with 15 mL of 2 mol/L HCl. After centrifugation at 3000 rpm for 15 min, the supernatant was collected and 10 mL of *n*-hexane was added, thoroughly mixed, and extracted 3 times to ensure it was defatted. The aqueous layer was extracted 5 times with 10 mL of ethyl acetate. The ethyl acetate extracts were dried in a rotary evaporator. Finally, the dry powder was dissolved in 1 mL of methanol and stored at −80 °C.

### 2.6. Instrumentation and Chromatographic Conditions

The identification of compounds was performed using UPLC-ESI-QTOF-MS/MS. Mobile phase: A, water and B, acetonitrile. Profile gradient: 0 min, 95% A.; 2 min, 95% A.; 25 min, 50% A.; 35 min, 5% A.; 37 min, 5% A.; 40 min, 95% A. The injection volume was 3 μL. The column was maintained at 35 °C, and the elution flow rate was maintained at 0.3 mL/min. The data were collected at 280 nm for the polyphenols. The 6538 QTOF was run in positive and negative ion mode (ESI) with scan range of *m*/*z* 100–1500. GS1: 55 psi; GS2: 55 psi; CUR: 35 psi. Other parameters included an ion source temperature (TEM): 550 °C (negative) and ion source voltage (IS): −4500 V (negative). Level 1 scan: Decluster voltage (DP): 100 V, focus voltage (CE): 10 V; secondary scan: TOF MS Product Ion IDA mode was used to collect mass spectrometry data; CID energy was 20, 40, and 60 V before injection; and the mass axis was corrected by CDS pump, in order that the mass axis error was less than 2 ppm. UPLC column: ZOBAX SB-C18 analytical column (ACQUITY UPLC, 5 μm, 50 × 4.6 mm, Waters Corp, Milford Sound, MA, USA).

### 2.7. Phenolic Content Measurement

The phenolic content of the FPs and BPs was determined using the Folin−Ciocalteu method [18] with some modifications. Briefly, 100 μL of sample was mixed with 800 μL ddH_2_O. Subsequently, 100 μL of 0.5 mol/L Folin−Ciocalteu was added and the mixture was incubated for 3 min. Next, 200 μL of 7% Na_2_CO_3_ solution was added. After incubating in a water bath at 30 °C for 2 h in the dark, the absorbance at 760 nm was measured. The phenolic content was expressed as gallic acid equivalents (mg GAE/100 g DW). 

### 2.8. Antioxidant Capacity Assay

The antioxidant activity of FPs and BPs was measured using 2,2-diphenyl-1-picrylhydrazyl (DPPH) and the ferric reducing antioxidant power (FRAP) assay.

DPPH radical scavenging activity assay: 0.1 mL of sample was added to 3.9 mL of 60 mmol/L DPPH solution. The mixture was maintained at 25 °C for 2 h in the dark and the absorbance was measured at 515 nm. The activity was expressed as milligrams Trolox per 100 g dry weight (mg Trolox/100 g DW).

FRAP assay: 0.1 mL of sample was mixed with FRAP solution at a ratio of 1:9. Then, the absorbance at 593 nm was recorded. Total FRAP was expressed as milligrams Trolox per 100 g dry weight (mg Trolox/100 g DW).

### 2.9. Statistical Analysis

Statistical analysis was performed for three independent replicates. The data were analyzed using Tukey’s significance test and Pearson correlation coefficients were calculated to determine the relationship between phenolic content and antioxidant capacity. SPSS version 21.0 software was used and the data are presented as the mean ± standard deviation.

## 3. Results and Discussion

**Figure 1:**
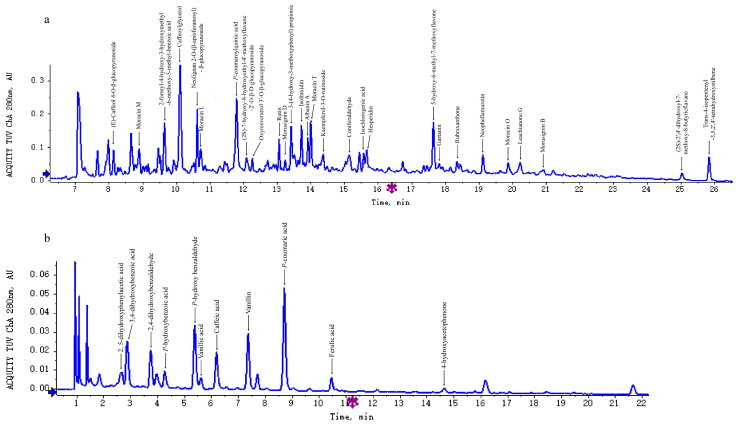
Representative UPLC chromatogram of FPs (**a**) and BPs (**b**) at 280 nm.

FPs and BPs were extracted by methanol and ethyl acetate, respectively and analyzed using UPLC-ESI-QTOF-MS/MS for the identification of the compounds. The phenolics were identified based on the retention time, molecular formula, *m*/*z,* and mass spectra fragmentation. A total of 39 phenolics, including 28 FPs and 11 BPs, were tentatively identified (Figure 1) and their UPLC chromatograms can be found in Figures S1 and S2. Among these, five phenolics were previously not reported in the mulberry plant. Moreover, the phenolic content and antioxidant capacity of FPs and BPs were measured, which indicated the significant antioxidant capacity of the mulberry seeds and the potential for phenolics extraction.

### 3.1. Identification of FPs from Mulberry Seeds

Table 1 lists the identification of 28 FPs in mulberry seeds, which were divided into the following groups: Flavonoids, phenolic acids and their derivatives, 2-arylbenzofurans, xanthone, stilbenes, coumarin derivatives, and other phenolics. Among these, five phenolics were reported for the first time including (E)-Caffeol 4-*O*-β-glucopyranoside, 2-formyl-4-hydroxy-3-hydroxymethyl-6-methoxy-5-methyl-benzoic acid, Neolignan 2-*O*-(β-apiofuranosyl)-β-glucopyranoside, Rubraxanthone, and Neophellamuretin.

#### 3.1.1. Flavonoids

According to Table 1, for compound **10** (tR = 13.04 min) with a molecular ion [M-H]^−^ at *m*/*z* of 609.14 and compound **19** (tR = 15.64 min) with a molecular ion at *m*/*z* of 609.18, both produced an MS^2^ fragment at an *m*/*z* of 301, which corresponded to quercetin and resulted from the loss of rutinoside. Therefore, these two compounds were flavanols with quercetin as the mother nucleus. Compound **10** was tentatively identified as rutin and compound **19** was identified as hesperidin when compared with an authentic standard. For compound **14** (tR = 13.90 min), the MS yielded a molecular ion [M-H]^−^ at *m*/*z* 353.10 and MS^2^ yielded two fragments at *m*/*z* 338.08 and 279.06. The molecular formula was C_20_H_18_O_6_. According to studies from other groups, compound **14** was tentatively identified as Albanin A [19]. Compound **16** (tR = 14.34 min), with a molecular formula of C_27_H_30_O_15_, produced a molecular ion [M-H]^−^ at *m*/*z* 593.15 and two fragments at *m*/*z* of 285.04 and 255.03. The fragment at *m*/*z* 285 was kaempferol, which is formed by the loss of glycoside, and the fragment at *m*/*z* 255 was a compound with a mother nucleus of flavonoid glycoside. Therefore, compound **16** was tentatively identified as kaempferol-3-*O*-rutinoside [20]. Compound **20** (tR = 17.63 min) with the formula C_17_H_14_O_4_ yielded a fragment ion [M-H]^−^ at *m*/*z* 281.08. MS^2^ yielded two fragments at *m*/*z* of 266.06 and 237.05. The fragmentation indicated that the compound lost a methyl group from the benzene ring to form *m*/*z* 266 [M-H-12]^−^ and continued to lose a methoxyl group to form *m*/*z* 237 [M-H-12-30]^−^. Compound **20** was tentatively identified as 5-hydroxy-6-methyl-7-methoxyflavone. 

Compound **23** (tR = 19.10 min) was tentatively identified as Neophellamuretin based on the molecular ion ([M+H^+^]) at *m*/*z* of 357.13 and two main fragments at *m*/*z* 325.10 [M+H-2OH]^+^ and 253.09 [M+H-C5H10O2]^+^. Neophellamuretin was found in *Epimedium koreanum* Nakai [21] and *Desmodium caudatum* [22]. This is the first time that Neophellamuretin was found to be present in mulberry seeds or other mulberry matrices.

Compound **25** (tR = 20.21 min) lost a hydroxyl to form *m*/*z* 339 and continued to lose two hydroxyls to form *m*/*z* 307. The remainder cracked into fragments at *m*/*z* 247 and 93. According to the MS fragments, compound **25** was tentatively identified as Leachianone G. Compound **27** (tR = 25.07 min) was a flavane which was first identified in mulberry leaves [23]. It yielded a molecular ion [M+H]^+^ at *m*/*z* of 359.15. MS^2^ = *m*/*z* 327.12 [M+H-OCH3]^+^ and was obtained through the loss of a methoxyl group and continued to lose butyric acid to form *m*/*z* 240.08 [M+H-OCH3-C4H7O2]^+^. Therefore, compound **27** was tentatively identified as (2S)-2′, 4′-dihydroxyl-7-methoxy-8-butyricflavane.

#### 3.1.2. Phenolic Acids and Their Derivatives

Phenolic acids are one group of aromatic secondary plant metabolites that exist widely in plants and exhibit a variety of physiological functions. Many phenolic acids and their derivatives, such as *P*-coumaroylquinic acid and isochlorogenic acid, have been reported in mulberry leaves and other mulberry matrices [24]. Two types of phenolic acid derivatives of benzoic acid and derivatives of cinnamic acid have been identified in mulberry seeds. 

Compounds **5** and **12** are derivatives of cinnamic acid. There was a molecular ion [M-H]^−^ at *m*/*z* 551.18 for compound **5** (tR = 10.60 min) and its fragmentation occurred when the mother nucleus lost hexose to form *m*/*z* 389 [M-H-162]^−^, which then cracked into *m*/*z* 341 [M-H-162-30]^−^ and *m*/*z* 193 corresponding to ferulic acid. Therefore, the compound was tentatively identified as Neolignan 2-*O*-(β-apiofuranosyl)-β-glucopyranoside, and it was first described in mulberry matrices. Compound **12** (tR = 13.40 min) with a molecular ion [M-H]^−^ at *m*/*z* 195.07 lost a methoxyl group and yielded an *m/z* of 165 [M-H-30]^−^. MS^2^ = *m*/*z* 150 [M-H-30-15]^−^ was formed by the loss of a hydroxyl group. The compound was tentatively identified as 3-(4-hydroxy-3-methoxyphenyl) propionic acid. The fragment at *m*/*z* 179 corresponded to caffeic acid, indicating that both compounds **4** and **18** were derivatives. Compound **4** was tentatively identified as caffeoylglycerol based on a precursor ion [M-H]^−^ at *m*/*z* 253.07 and other fragments at *m*/*z* 161.02 and 135.03. Another fragment of compound **18** (tR = 15.54 min) yielded an *m*/*z* of 191, which was consistent with quininic acid and compound **18** was identified with an authentic standard as isochlorogenic acid. For compound **7** (tR = 11.75 min) with a molecular ion [M-H]^−^ at *m*/*z* 337.15, there was also a fragment that matched quininic acid at *m/z* 191.05 and it was tentatively identified as *P*-coumaroylquinic acid, which belongs to derivatives of coumaric acid. Compound **5** belonged to derivatives of benzoic acid. It produced a molecular ion [M-H]^−^ at *m*/*z* 239.05, two MS^2^ fragments at *m*/*z* 209.04 and 150.03. This compound was tentatively identified as 2-formyl-4-hydroxy-3-hydroxymethyl-6-methoxy-5-methyl-benzoic acid.

#### 3.1.3. 2-Arybenzofuran Derivatives

The 2-arybenzofuran and its derivatives present a series of isoprenoid-substituted phenolic compounds and the Morus species has been regarded as a rich source. Moracin families are a type of 2-arybenzofuran derivative with the basic structure of benzofuran heterocycle and four compounds were identified in mulberry seeds. Compounds **2**, **6**, **15**, and **25** were characterized as Moracin.

Compound **4** (tR = 8.86 min) with a molecular ion [M-H]^−^ at *m*/*z* 241.09 yielded a fragment ion at *m*/*z* 226.06 after a loss of a hydroxyl group and it was tentatively identified as Moracin M. Compound **6** (tR = 10.71 min) was tentatively identified as Moracin L based on a molecular ion [M+H]^+^ at *m*/*z* of 325.10 and a fragment ion at *m*/*z* 307 resulting from the loss of a hydroxy group. For compound **15** (tR = 13.98 min), the presence of a fragment ion [M+H]^+^ at *m*/*z* of 341.14 suggested that the molecular mass was 340. MS^2^ yielded three fragments at *m*/*z* of 309.11, 137.06, and 161.05. The fragmentation indicated that a precursor ion lost a methoxyl group to form *m*/*z* 309 and subsequently cracked into *m*/*z* 161 and 137. Therefore, it was identified as Moracin T. Moracin O was tentatively identified according to the molecular ion at *m*/*z* 325.10 and two MS^2^ fragments at *m*/*z* 310.08 and 241.05 of compound **25** (tR = 19.86 min).

#### 3.1.4. Xanthone Derivatives

Xanthone and its derivatives comprise an important part of natural phenolics with a basic dibenzo-γ-pirone scaffold. Four new xanthone derivatives (compounds **11**, **21**, **22**, and **26**) were isolated from mulberry seeds.

Four xanthone derivatives including Morusignins A, B, C, and D have been isolated from the root bark of *Morus insignis* Bur. 1 [25], and Morusignins B and D were found in mulberry seeds. Compound 11 (tR = 13.22 min) showed a molecular ion [M-H]^−^ at *m*/*z* 341.10 and two fragments at *m*/*z* 326 [M-H-15]^−^ and *m*/*z* 267 [M-H-74]^-^. Compound **26** (tR = 20.90 min) showed a molecular ion [M-H]^−^ at *m*/*z* 327.09 and two fragments at *m*/*z* 312 [M-H-15]^−^ and *m*/*z* 96 [M-H-30]^−^. By comparison with previous studies, compounds **11** and **26** were tentatively identified as Morusignins B and D, respectively. Compound **21** (tR = 17.80 min) was a polyphenolic isoprenylated xanthone in mulberry seeds. According to its molecular ion [M+H]^+^ at *m*/*z* 397.17 and two MS^2^ fragments at *m*/*z* 137 and 273, it was tentatively identified as Gartanin. Rubraxanthone was isolated from mulberry matrices for the first time. The presence of an *m*/*z* 311.09 [M-H]^−^ suggested that the molecular mass of compound **22** (tR = 18.84 min) was 312. Its fragmentation produced two fragments at *m*/*z* 296 [M-H-15]^−^ and *m/z* 253 [M-H-58]^−^. Compared with previous studies, the compound was tentatively identified as Rubraxanthone. 

#### 3.1.5. Stilbenes

Two stilbenes were isolated from mulberry seeds. First, a molecular ion [M-H]^−^ at *m*/*z* 405.11 was found with two fragments at *m*/*z* 243 and 211, which corresponded to the loss of Glu and two hydroxyls, respectively. Therefore, compound **9** (tR = 12.24 min) was tentatively identified as Oxyresveratrol 3′-*O*-β-glucopyranoside. Another derivative of resveratrol—Trans-4-isopentenyl-3,5,2′,4′-tetrahydroxystilbene was identified. Compound **28** (tR = 25.80 min) yielded a molecular ion [M-H]^−^ at *m*/*z* 311.12 and two MS^2^ fragments at *m*/*z* 293.11 and 241.04. Based on previous studies, the structure of compound 28 was tentatively determined.

#### 3.1.6. Coumarin Derivatives

Based on the analysis of UPLC-ESI-QTOF-MS/MS, compound **13** (tR = 13.70 min) was identified as Isofraxidin which was a prominent hydroxy coumarin. The MS showed a molecular ion [M-H]^−^ at *m*/*z* 221.04. The molecule yielded a fragment at *m/z* 162.03 (loss of two methoxyl groups) and subsequently cracked to form *m*/*z* 134 [M-H-2(-OCH3)-28]^−^. Based on ions from fragmentation and compared with an authentic standard, compound **13** was identified as Isofraxidin. 

#### 3.1.7. Other Phenolics

There were three other phenolics (compounds **1**, **8**, and **17**) isolated from mulberry seeds. The formula of compound **1** (tR = 8.11 min) was C_15_H_20_O_8_. The MS yield of the molecular ion [M-H]^−^ at *m*/*z* 327.10 and MS^2^ = *m*/*z* 147.04 was obtained by a loss of glycoside. Therefore, compound 1 was tentatively identified as (E)-caffeol 4-*O*-β-glucopyranoside and this is the first report of its presence in mulberry seeds. Compound **10** (tR = 12.07 min) exhibited a molecular ion [M-H]^−^ at *m*/*z* 477.18, the fragment of MS^2^ yielded an *m*/*z* of 315 (loss of glycoside), and the compound was tentatively identified as (2S)-7-hydroxy-8-hydroxyethyl-4′-methoxyflavane-2′-*O*-β-d-glucopyranoside. Compound **17** (tR = 15.22 min) produced a molecular ion [M-H]^−^ at *m*/*z* 177.06 and two MS^2^ fragments at *m*/*z* 162.03 and 134.04. The fragment at *m*/*z* 162 was obtained by the loss of a methyl group and *m*/*z* 134 was formed by the loss of aldehyde. The compound was identified as coniferaldehyde using an authentic standard.

### 3.2. Identification of BPs in Mulberry Seeds

Numerous FPs in mulberry seeds have been reported; however, there have not been many studies focused on BPs from mulberry seeds. Therefore, our study will complement the existing knowledge of BPs in mulberry seeds. BPs accounted for an average of 24% of the total phenolics in foods, such as fruits and vegetables, and many bioactive properties have been reported including antioxidant, anti-inflammatory, and hepatoprotective activities [26]. As a result, BPs in food are of significant biological importance for their high content and biological activity. In this study, a total of 11 BPs in mulberry seeds were isolated (Table 1), which were primarily phenolic acids.

#### 3.2.1. Phenolic Acids and Their Derivatives

Six phenolic acid derivatives were identified in the form of BPs from mulberry seeds. Compounds **2**, **4**, and **6** were identified as derivatives of benzoic acids and compounds **7**, **9**, and **10** were identified as derivatives of cinnamic acids with authentic standards.

Compound **2** (tR = 2.86 min) produced a molecular ion [M-H]^−^ at *m*/*z* 153.02. The presence of carboxyl and hydroxyl groups in the compound was inferred from two MS^2^ fragments at *m*/*z* 109.03 and 91.01. Therefore, compound **2** was identified as 3,4-dihydroxybenzoic acid. Compound **4** (tR = 4.26 min) exhibited a molecular ion [M-H]^−^ at *m*/*z* 137.03 and a fragment at *m*/*z* of 93.03. The main fragment at *m*/*z* 93.03 [M-CO2-H] ^−^ in MS^2^ resulted from the loss of a carboxyl group and the compound was identified as *P*-hydroxybenzoic acid. Compound **6** (tR = 5.60 min) was identified as vanillic acid based on its fragmentation. The precursor ion [M-H]^−^ at *m*/*z* 167.04 lost a hydroxyl group to yield the MS^2^ fragment of *m*/*z* 152.01, then subsequently lost a carboxyl group to form a fragment at *m*/*z* 108.02. 

There was a similar structure of C6-C3 in compounds **7**, **9**, and **10**; therefore, they may be classified as cinnamic acid derivatives. Compound **7** (tR = 6.18 min) exhibited a molecular ion [M-H]^−^ at *m*/*z* 179.03, a loss of a carboxyl group [44 Da] resulted in a fragment of *m*/*z* 135.04, which was identified as caffeic acid from previous reports. The molecular ion [M-H]^−^ of compound **9** was at *m*/*z* 163.04, 16 Da smaller than the precursor ion of caffeic acid [179 Da], which indicated the lack of a hydroxyl group. With respect to the other two fragments at *m*/*z* 119.05 and 93.05, compound **9** was identified as *P*-coumaric acid. For compound **10** (tR = 10.45 min), the MS yielded a molecular ion [M-H]^−^ at *m*/*z* 193.05 and MS^2^ showed two fragments at *m*/*z* 178.02 and 134.03; thus, it was identified as ferulic acid.

#### 3.2.2. Other Phenolics

Compound **1** (tR = 2.66 min) yielded a molecular ion [M-H]^−^ at *m*/*z* 167.04 and MS^2^ yielded a fragment of *m*/*z* 123.04 resulting from the loss of a carboxyl group. The compound was tentatively identified as 2,5-dihydroxyphenylacetic acid. The molecular ion [M-H]^−^ of compound **3** (tR = 3.74 min) appeared at *m*/*z* 137.03 and its formula was C_7_H_6_O_3_, which was consistent with compound **4** (*P*-hydroxybenzoic acid). However, the fragments in the second-order mass spectrum were different, which suggested that they were isomers. The fragment of compound **3** at *m*/*z* 108 was formed by the loss of an aldehyde group from the precursor ion; thus, the compound was tentatively identified as 2,4-dihydroxybenzaldehyde. For compound **5** (tR = 5.37 min), there was a 29 Da difference between the molecular weights of the fragment at *m*/*z* 121 and the precursor ion [M-H]^−^ at *m*/*z* 92.03, which corresponded to a loss of an aldehyde group. Therefore, compound **5** was tentatively identified as *P*-hydroxy benzaldehyde based on published reports. Compound **8** (tR = 7.36 min) produced a molecular ion [M-H]^−^ at *m*/*z* 151.00 and two MS^2^ fragments at *m/z* 123.01 and 107.01. The fragment at *m*/*z* 123.01 resulted from the loss of an aldehyde group and the molecular weight difference of 30 Da between the two fragments matched with a methoxyl group. Finally, compound **8** was identified as vanillin after comparing it with an authentic standard. Compound **11** (tR = 14.63 min) showed a molecular ion [M-H]^−^ at *m*/*z* 137.06, which was consistent with compound **3** (2,4-dihydroxybenzaldehyde) and compound **4** (*P*-hydroxybenzoic acid). Their molecular formulas and fragments in the second-order mass spectrum were different, which indicated that compound **11** was not an isomer of compounds **3** and **4**. On the basis of the two MS^2^ fragments at an *m*/*z* of 93.06 and 77.04, compound **11** was tentatively identified as 4-hydroxyacetophenone.

### 3.3. Phenolic Content of FPs and BPs from Extracts

The phenolic content of FPs and BPs in mulberry seeds from six varieties was quantified using the Folin−Ciocalteu method and the results are presented in Table 2. The phenolic content of FPs ranged from 76.104 mg GAE/100 g DW to 109.107 mg GAE/100 g DW. 

The higher phenolic content of the FPs was observed in Guiyou 12 and Guiyou 62. The lower phenolic content of FPs was in Shisheng, Yue 69851 and Yu 711, which was at a significantly lower level compared with the other groups. For BPs, the phenolic content of ethyl acetate extracts ranged from 38.041 mg GAE/100 g DW to 44.973 mg GAE/100 g DW, which was much lower compared with the FP content. The lowest phenolic content of the ethyl acetate extracts was observed in Shisheng, whereas the others were above 40 mg GAE/100 g DW. Only the difference between Shisheng and the other five varieties was significant. BPs may be released by the presence of intestinal flora to provide large amounts of biological activities in vivo, thus more treatments should be developed to promote the release [27]. As a result, 63.4–70.8% of phenolics in mulberry seeds of different varieties existed in free form and 29.2–36.7% of phenolics existed in bound form. Therefore, phenolics in mulberry seeds were mainly in a free form as they contained approximately 67.5% FPs and 32.5% BPs on average. This finding was consistent with earlier studies that reported FPs accounted for the majority of phenolics in common fruits, vegetables, and seeds [28,29]. 

Mulberry matrices including fruits, leaves, and other parts are a valuable source of phenolics. Mulberry leaves are rich in phytochemical compounds, and the total phenolic content (TPC) ranges from 12.81 to 16.13 mg GAE/g DW [30]. Butkhup et al. [31] found that the average TPC range for white mulberry fruits was 104.78–213.53 mg GAE/100 g DW. It can be seen in Table 2 that free and bound phenolic content in mulberry seeds reached the lower limit for fruits. Seed coat consisted of epidermis, chlorenchyma, parenchyma, and other cells, and these cells all contain organs, such as vesicles and cell walls, which are the main locations of free and bound phenolics, respectively [32]. Therefore, seed contained high amounts of FPs and BPs. However, few studies have reported the TPC of mulberry seeds. Gómez-Mejía et al. [13] extracted phenolic compounds with a hydroethanolic solution (80:20 *v*/*v* ethanol–water), and total phenolic compounds in mulberry seed extracts were determined to be 8.02 mg/g by HPLC-DAD-MS analysis. However, Kim et al. [33] reported that TPC of the MeOH extract of Morus alba seeds was 367.26 mg GAE/100 g DW, which was considerably higher than our results. The variation of TPC in mulberry seeds as well as other mulberry matrices, was attributable to many factors, such as genetic differences in the varieties, physiological state, harvest time, and environmental parameters [34].

In this study, the variety and environment are the predominant factors. Shisheng is a wild mulberry variety without artificially breeding; therefore, it showed the lowest phenolic content of FPs and BPs, which was significantly lower compared with the elite hybrid varieties. Yu 711 is a diploid mulberry variety bred from Yu 54 and Yu 2, and was primarily planted in the lower reaches of the Yangtze River in a temperate climate. The remaining four, Guiyou 12, Guiyou 62, Teyou 2, and Yue 69851 are from the Guangdong and Guangxi Province in China, regions with a subtropical humid monsoon climate. Among these, three varieties from Guangxi Province exhibit a closer genetic relationship due to their parents: Guiyou 12 is a diploid bred from Sha 2 and Gui 7722, Guiyou 62 is a diploid bred from 7862 and Gui 7722, Teyou 2 is a triploid bred from 7862 and Guiyou P58. Consequently, the close genetic relationship, growth environment, and artificial selection have provided them with a higher and similar FP and BP content. Similar results were observed by Zou et al. [35] in which they reported that the phenolic content of mulberry leaves significantly varied between different cultivars and collection months. With respect to TPC, Guiyou 12, Guiyou 62, and Teyou 2 are present with the highest phenolic content of FPs and BPs, which enable them to produce more potential varieties.

### 3.4. Antioxidant Capacity of Extracts

The antioxidant capacities of FPs and BPs from six varieties of mulberry seeds were evaluated using two methods: DPPH and FRAP. The reagent, 1,1-diphenyl-2-picrylhydrazyl (DPPH), can capture hydrogen ions and FRAP is based on the reduction of ferroin analogs [36]. The results are shown in Table 3.

#### 3.4.1. DPPH Radical Scavenging Activity and FRAP

From the DPPH assay, the antioxidant activity of FPs (78.85–105.46 mg Trolox/100 g DW) from all varieties was considerably higher compared with the antioxidant activity of BPs (41.84–55.08 mg Trolox/100 g DW). The highest antioxidant activity of FPs was observed in three varieties: Teyou 2, Guiyou 62, and Yue 69851, while the results of other three varieties were below 95 mg Trolox/100 g DW. The higher antioxidant activity for the BPs was observed in Guiyou 12 and Guiyou 62, whereas the lowest activity for the BPs was observed in Shisheng. The activities of the other varieties were similar and showed significant differences.

From the FRAP test, the antioxidant activity of FPs ranged from 60.31 mg Trolox/100 g DW to 75.87 mg Trolox/100 g DW. The highest antioxidant activity of FPs was observed in Guiyou 12, whereas the lowest activity was in Shisheng, and other varieties were slightly different and showed no significant differences. The antioxidant activity of the BPs was considerably lower compared with FPs. The highest antioxidant activity for the BPs was Yue 69851, followed by Guiyou 62 and Guiyou 12, and the activity of Shisheng and Teyou 2 was significantly lower than other varieties. 

Kim et al. [33] reported that methanol extracts of mulberry seed exhibited high radical scavenging activity against DPPH (IC_50_ = 0.15 mg/mL). Gómez-Mejía et al. [13] measured the antioxidant activity of mulberry seed extracts using the thiobarbituric acid reactive substance assay and oxidative hemolysis inhibition assay (OxHLIA). The IC_50_ for mulberry seeds was 23 ± 2 µg/mL, indicating the significantly better antioxidant capacity of mulberry seeds compared with grape seeds (IC_50_ = 168 ± 3 µg/mL) and mulberry fruit ethanol extracts (EC_50_ > 100 μg/mL). In addition, OxHLIA in mulberry seeds exhibited a higher anti-haemolytic activity. 

Overall, the FPs and BPs of Guiyou 12, Guiyou 62, and Teyou 2 exhibited higher DPPH radical scavenging activity and ferric reducing antioxidant power. The extracts of the mulberry seed may act as packaging materials to inhibit lipid peroxidation of food, given its high anti-oxidative effects [37].

#### 3.4.2. Relationship between Antioxidant Capacity and TPC

Phenolic compounds contribute significantly to the antioxidant capacity of plants. The relationship between phenolic compound content and antioxidant function has attracted considerable attention and many researchers have confirmed a linear correlation between the two [38]. However, this is not applicable to all phenolic content and their associated antioxidant effects in mulberry seeds. 

For example, as shown in Table 2 and Table 3, the FP content of Guiyou 12 was highest, whereas the DPPH radical scavenging activity of Teyou 2 was highest, but the *r*^2^ value of the Pearson correlation was 0.40, indicating a relatively poor correlation between them (Table 4). In contrast, there was a higher correlation coefficient between FPs and FRAP, BPs and DPPH, BPs and FRAP, and their high phenolic content was associated with a high antioxidant capacity. The corresponding *r*^2^ values were 0.76 (*p* < 0.01), 0.07 (*p* < 0.01), and 0.58 (*p* < 0.05), respectively, which suggests that a significant correlation exists between phenolic content and antioxidant capacity. The results showing a weak or significant correlation was consistent with previous studies. Babbar et al. [39] reported that the *r*^2^ value of TPC extracted from six kinds of fruit residues and their corresponding DPPH radical scavenging activity, ABTS radical scavenging activity, and reducing power was 0.36, 0.49, and 0.66, respectively. Karamać et al. [40] reported that there was no significant correlation between TPC of white lupin seed extracts and DPPH scavenging activity. These results demonstrated an insignificant relationship. 

Since high FP or BP content did not correspond with high antioxidant activity, there may be non-phenolic compounds including ascorbates, terpenes, and pigments that contributed to the antioxidant function; different compounds in FPs and BPs showed varying levels of antioxidants [39]. The antioxidant activity of phenolics was greatly influenced by chemical structures of compounds, especially the number and position of aromatic and hydroxyl groups, and it has been proven to be closely related to the degree of hydroxylation [41]. For example, flavonoids and phenolic acids with more hydroxyl groups tended to exhibit stronger antioxidant activity than others. Arruda et al. [42] found that flavonoids were the main source of antioxidant activity in the Araticum pulp and peel, while phenolic acids, such as ferulic acid, caffeic acid, and chlorogenic acid contributed the most to the antioxidant activity of seeds. Our results showed that there were eight kinds of flavonoids, six kinds of phenolic acids in FPs, and only six kinds of phenolic acids in BPs; therefore, different phenolics contribute to antioxidant activity differently and brought about the various correlation between phenolic content and antioxidant activity. In addition, the antagonistic and synergistic reactions between phenolics and other chemicals may result in the poor correlations [43]. As a result, the antioxidant capacity of non-phenolics and reactions between phytochemicals require further studies to better utilize plant byproducts, such as mulberry seeds.

## 4. Conclusions

Phenolic compounds from six varieties of mulberry seeds were extracted and identified in free and bound forms. The content of FPs and BPs was measured and their corresponding antioxidant capacity was determined. The results of UPLC-ESI-QTOF-MS/MS revealed 28 FPs, 11 BPs, and 5 FPs including (E)-caffeol 4-*O*-β-glucopyranoside, 2-formyl-4-hydroxy-3-hydroxymethyl-6-methoxy-5-methyl-benzoic acid, Neolignan 2-*O*-(β-apiofuranosyl)-β-glucopyranoside, Rubraxanthone, and Neophellamuretin, which were first reported in mulberry matrices compared with previous studies. For the phenolic content and antioxidant capacity, Guiyou 12, Guiyou 62, and Teyou 2 were rich in both FPs and BPs. FPs of Teyou 2 displayed the highest DPPH radical scavenging activity, whereas BPs of Guiyou 12 exhibited the highest DPPH radical scavenging activity. Guiyou 12 showed the highest FRAP in FPs, whereas Guiyou 62 contained the highest FRAP for the BPs. All three showed higher DPPH radical scavenging activity and FRAP compared with the other varieties. The results revealed that there was a correlation to a certain extent between the two; however, further studies are essential in this field. Consequently, our study was the first comprehensive analysis of FPs and BPs in mulberry seeds to date and five new free phenolics were identified in the mulberry seeds for the first time. The results provide essential information for the identification and antioxidant capacity of FPs and BPs from mulberry seeds. As a byproduct of the food manufacturing industry, mulberry seeds are expected to be a source of bioactive compounds and Guiyou 12, Guiyou 62, and Teyou 2 are specially recommended for consideration.

## Figures and Tables

**Table 1:** Identification of FPs and BPs in mulberry seeds.

**Free Phenolics**											
**No**	**Retention Time (tR)/min**	**[M-H]^−^** **or [M+H]^+^**	**Fragment Ions**	**Formula**	**Identification**	**Varieties**					
						**Shisheng**	**Yu 711**	**Guiyou 12**	**Guiyou 62**	**Teyou 2**	**Yue 69851**
1	8.11	327.10	147.04	C_15_H_20_O_8_	(E)-Caffeol 4-*O*-β-glucopyranoside	+	+	+	+	+	+
2	8.86	241.09	226.06	C_14_H_10_O_4_	Moracin M	+	+	+	+	+	+
3	9.63	239.05	209.04, 150.03	C_11_H_12_O_6_	2-formyl-4-hydroxy-3-hydroxymethyl-6-methoxy-5-methyl-benzoic acid	+	+	+	+	+	+
4	10.08	253.07	179.03, 161.02	C_12_H_14_O_6_	Caffeoylglycerol	+	+	+	+	+	+
5	10.60	551.17	341.10, 193.05	C_26_H_32_O_13_	Neolignan 2-*O*-(β-apiofuranosyl)-β-glucopyranoside	+	+	+	+	+	+
6	10.71	325.12	307.13, 191.08	C_19_H_16_O_5_	Moracin L	+	+	+	+	+	+
7	11.75	337.15	191.05	C_16_H_18_O_8_	*P*-coumaroylquinic acid	+	+	+	+	+	+
8	12.07	477.18	462.15, 315.12	C_24_H_30_O_10_	(2S)-7-hydroxy-8-hydroxyethyl-4′-methoxyflavane-2′-*O*-β-d-glucopyranoside	+	+	+	+	+	+
9	12.24	405.11	211.06, 163.03	C_20_H_22_O_9_	Oxyresveratrol 3′-*O*-β-glucopyranoside	+	+	+	+	+	+
10	13.04	609.14	301.03	C_27_H_30_O_16_	Rutin *	+	+	+	+	+	+
11	13.22	341.10	326.08, 267.06	C_19_H_18_O_6_	Morusignin D	+	+	+	+	+	+
12	13.40	195.07	165.06, 150.03	C_10_H_12_O_4_	3-(4-hydroxy-3-methoxyphenyl) propionic acid	+	+	+	+	+	+
13	13.70	221.04	162.03, 134.04	C_11_H_10_O_5_	Isofraxidin *	+	+	+	+	+	+
14	13.90	353.10	338.08, 279.06	C_20_H_18_O_6_	Albanin A	+	+	+	+	+	+
15	13.98	341.14	309.11, 137.06	C_20_H_20_O_5_	Moracin T	+	+	+	+	+	+
16	14.34	593.15	285.04, 255.03	C_27_H_30_O_15_	Kaempferol-3-*O*-rutinoside *	+	+	+	+	+	+
17	15.22	177.06	162.03, 134.04	C_10_H_10_O_3_	Coniferaldehyde *	+	+	+	+	+	+
18	15.54	515.12	353.08, 179.03	C_25_H_24_O_10_	Isochlorogenic acid *	+	+	+	+	+	+
19	15.64	609.18	301.07	C_28_H_34_O_15_	Hesperidin *	+	+	+	+	+	+
20	17.63	281.08	266.06, 237.05	C_17_H_14_O_4_	5-hydroxy-6-methyl-7-methoxyflavone	+	+	+	+	+	+
21	17.80	397.16	273.11, 137.06	C_23_H_24_O_6_	Gartanin	+	+	+	+	+	+
22	18.34	311.09	296.07, 253.05	C_18_H_16_O_5_	Rubraxanthone	+	+	+	+	+	+
23	19.10	357.13	325.10, 253.09	C_20_H_20_O_6_	Neophellamuretin	+	+	+	+	+	+
24	19.86	325.10	310.08, 241.05	C_19_H_18_O_5_	Moracin O	+	+	+	+	+	+
25	20.21	355.11	307.09, 247.07	C_20_H_20_O_6_	Leachianone G	+	+	+	+	+	+
26	20.90	327.09	312.05, 296.06	C_18_H_16_O_6_	Morusignin B	+	+	+	+	+	+
27	25.07	359.15	327.12, 240.08	C_20_H_22_O_6_	(2S)-2′,4′-dihydroxyl-7-methoxy-8-butyricflavane	+	+	+	+	+	+
28	25.80	311.13	241.04	C_19_H_20_O_4_	Trans-4-isopentenyl-3,5,2′,4′-tetrahydroxystilbene	+	+	+	+	+	+
**Bound Phenolics**											
**No**	**Retention Time (tR)/min**	**[M-H]^−^** **or [M+H]^+^**	**Fragment Ions**	**Formula**	**Identification**	**Varieties**					
						**Shisheng**	**Yu 711**	**Guiyou 12**	**Guiyou 62**	**Teyou 2**	**Yue 69851**
1	2.66	167.04	123.04	C_8_H_8_O_4_	2,5-dihydroxyphenylacetic acid	+	+	+	+	+	+
2	2.86	153.02	109.03, 91.01	C_7_H_6_O_4_	3,4-dihydroxybenzoic acid *	+	+	+	+	+	+
3	3.74	137.03	108.02	C_7_H_6_O_3_	2,4-dihydroxybenzaldehyde	+	+	+	+	+	+
4	4.26	137.03	93.03	C_7_H_6_O_3_	*P*-hydroxybenzoic acid *	+	+	+	+	+	+
5	5.37	121.03	92.03	C_7_H_6_O_2_	*P*-hydroxy benzaldehyde	+	+	+	+	+	+
6	5.60	167.04	152.01, 108.02	C_8_H_8_O_4_	Vanillic acid *	+	+	+	+	+	+
7	6.18	179.03	135.04	C_9_H_8_O_4_	Caffeic acid *	+	+	+	+	+	+
8	7.36	151.04	123.01, 107.01	C_8_H_8_O_3_	Vanillin *	+	+	+	+	+	+
9	8.70	163.04	119.05, 93.05	C_9_H_8_O_3_	*P*-coumaric acid *	+	+	+	+	+	+
10	10.45	193.05	178.02, 134.03	C_10_H_10_O_4_	Ferulic acid *	+	+	+	+	+	+
11	14.63	137.06	93.07, 77.04	C_8_H_8_O_2_	4-hydroxyacetophenone	+	+	+	+	+	+

+: Detected; *: Verified with authentic standards.

**Table 2:** Total phenolic content of FPs and BPs in mulberry seeds.

Varieties	FPs (mg GAE/100 g DW)	BPs (mg GAE/100 g DW)
Shisheng	76.10 ± 2.21 ^c^	38.04 ± 0.67 ^b^
Yu 711	81.23 ± 4.86 ^c^	43.53 ± 3.33 ^a^
Guiyou 12	109.11 ± 3.96 ^a^	44.97 ± 2.34 ^a^
Guiyou 62	105.07 ± 11.71 ^ab^	46.28 ± 0.32 ^a^
Teyou 2	96.94 ± 5.76 ^b^	44.81 ± 0.84 ^a^
Yue 69851	76.56 ± 3.32 ^c^	44.31 ± 1.14 ^a^

Values with the same letter are not significantly different (*p* > 0.05).

**Table 3:** Antioxidant capacity of FPs and BPs in mulberry seeds.

**Varieties**	**DPPH Radical Scavenging Activity (mg Trolox/100 g DW)**	
	**FPs**	**BPs**
Shisheng	78.85 ± 6.10 ^c^	41.84 ± 0.33 ^d^
Yu 711	84.88 ± 3.57 ^bc^	48.28 ± 1.73 ^bc^
Guiyou 12	94.64 ±2.36 ^ab^	55.08 ± 3.72 ^a^
Guiyou 62	103.83 ± 7.27 ^a^	50.93 ± 1.32 ^b^
Teyou 2	105.46 ± 4.98 ^a^	45.73 ± 0.89 ^c^
Yue 69851	101.02 ± 9.46 ^a^	47.46 ± 1.19 ^c^
**Varieties**	**Ferric Reducing Antioxidant Power** ** (mg Trolox/100 g DW)**	
	**FPs**	**BPs**
Shisheng	60.31 ± 1.27 ^c^	39.45 ± 0.99 ^d^
Yu 711	68.47 ± 1.19 ^a^	43.46 ± 1.07 ^bc^
Guiyou 12	75.87 ± 2.62 ^b^	44.22 ± 2.17 ^bc^
Guiyou 62	72.76 ± 2.62 ^b^	44.35 ± 1.31 ^b^
Teyou 2	70.73 ± 0.72 ^b^	41.79 ± 1.30 ^cd^
Yue 69851	68.99 ± 2.46 ^b^	47.38 ± 1.09 ^a^

Values with the same letter are not significantly different (*p* > 0.05).

**Table 4:** Correlation between TPC and antioxidant capacity of mulberry seeds.

Correlation	*r* ^2^
FPs-DPPH	0.40 ^ns^
FPs-FRAP	0.76 **
BPs-DPPH	0.70 **
BPs-FRAP	0.58 *

*: Correlation is significant (*p* < 0.05); **: Correlation is significant (*p* < 0.01); ^ns^: Correlation is not significant (*p* ≥ 0.05).

## Data Availability

Data is contained within the article and supplementary material.

## Supplementary Materials

The following supporting information can be downloaded at: https://www.mdpi.com/article/10.3390/antiox11091764/s1. Figure S1: UPLC chromatogram of FPs at 280 nm. A: Shisheng; B: Yu 711; C: Guiyou 12; D: Guiyou 62, E: Teyou 2; F: Yue 69851; Figure S2: UPLC chromatogram of BPs at 280 nm. A: Shisheng; B: Yu 711; C: Guiyou 12; D: Guiyou 62, E: Teyou 2; F: Yue 69851.
