# Supplementary material for: Identification and Antioxidant Capacity of Free and Bound Phenolics in Six Varieties of Mulberry Seeds Using UPLC-ESI-QTOF-MS/MS
Source: Antioxidants (Basel). 2022 Sep 7;11(9):1764. doi: 10.3390/antiox11091764 (PMC9495565; doi:10.3390/antiox11091764)
Supplement: Supplementary file 1 [file antioxidants-11-01764-s001.zip › antioxidants-1803713-supplementary.pdf]

Figure S1 UPLC chromatogram of FPs at 280 nm. A: Shisheng; B: Yu 711; C: Guiyou 12; D: Guiyou 62, E: Teyou 2; F: Yue 69851.

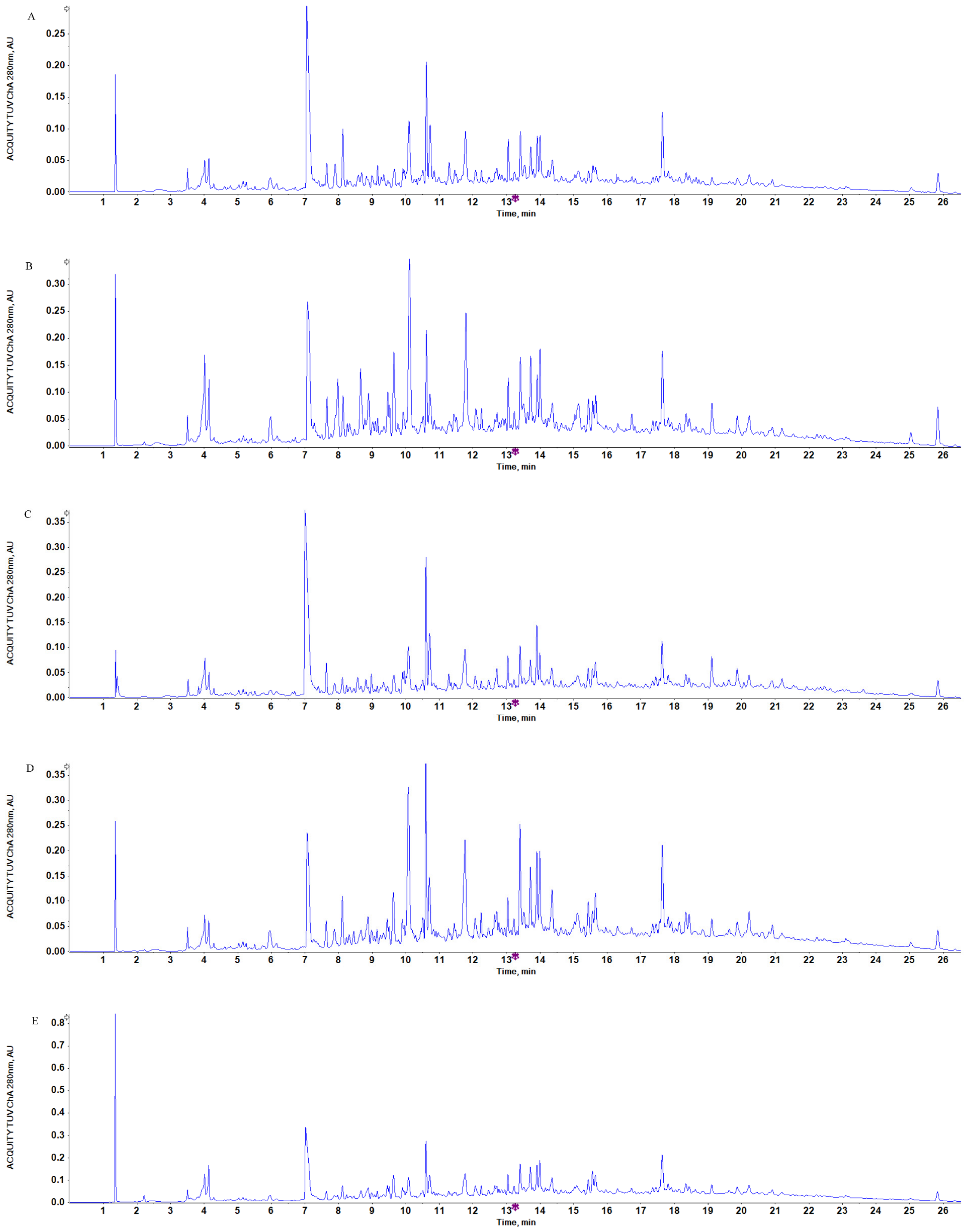

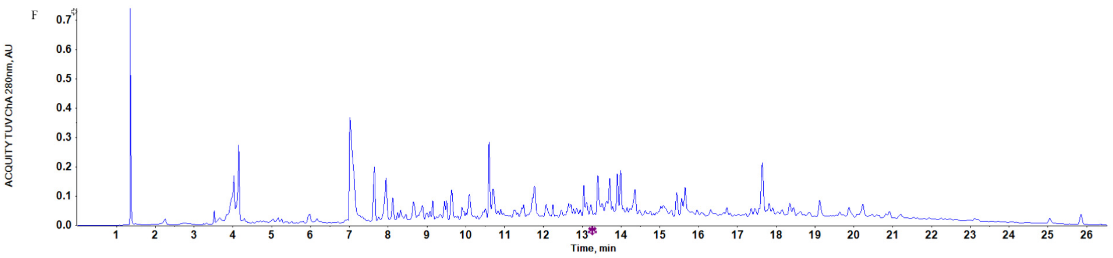

Figure S2 UPLC chromatogram of BPs at 280 nm. A: Shisheng; B: Yu 711; C: Guiyou 12; D: Guiyou 62, E: Teyou 2; F: Yue 69851.

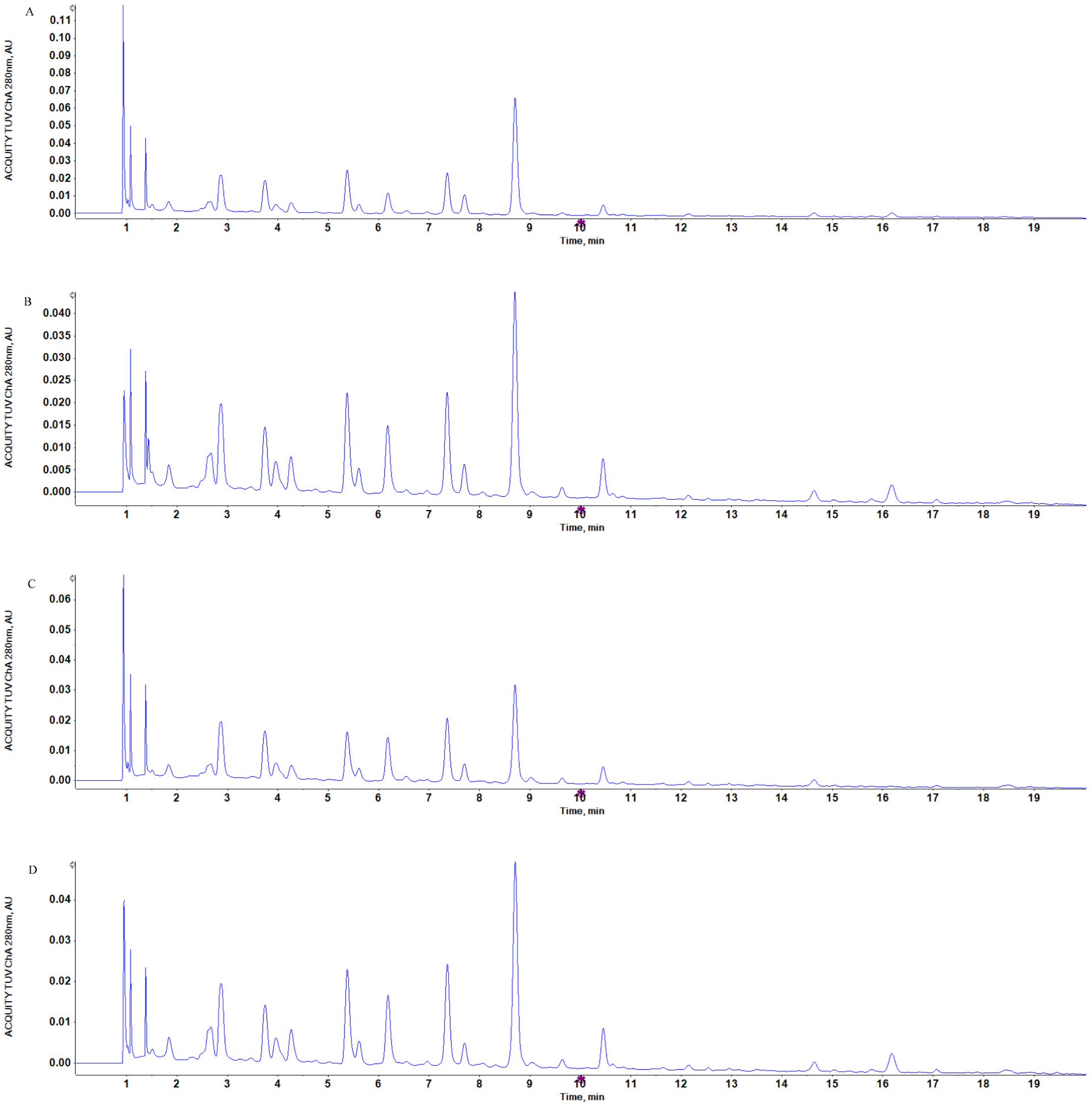

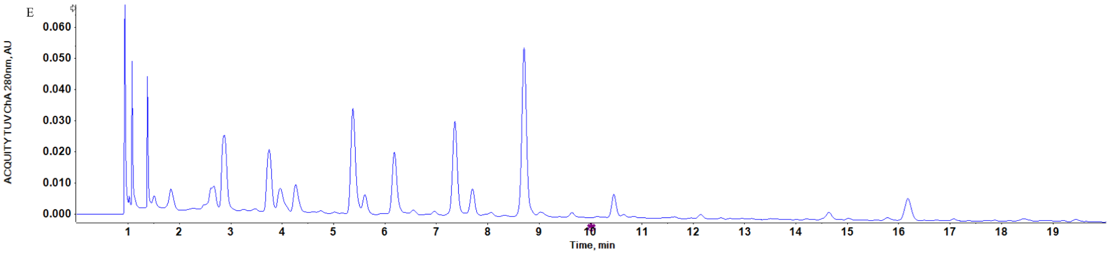
E
ACQUITY TUV ChA 280nm, AU
0.060
0.050
0.040
0.030
0.020
0.010
0.000
1 2 3 4 5 6 7 8 9 10 11 12 13 14 15 16 17 18 19
Time, min

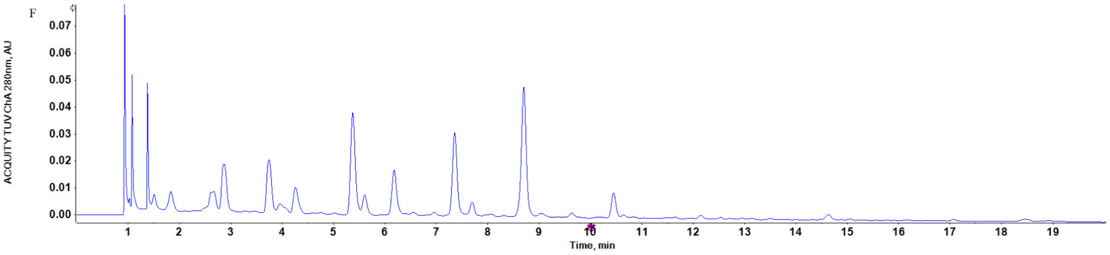
F
ACQUITY TUV ChA 280nm, AU
0.07
0.06
0.05
0.04
0.03
0.02
0.01
0.00
1 2 3 4 5 6 7 8 9 10 11 12 13 14 15 16 17 18 19
Time, min
